# Report on Vaccination

**Published:** 1846-02

**Authors:** Henry T. Child

**Affiliations:** Vaccine Physician, N. D. N. L.; Northern Liberties


*Report on Vaccination.* By Henry T. Child, M. D. Communicated for the Examiner.

To the Board of Commissioners of the Northern Liberties.

*Gentlemen*,--Since entering upon the duties of Vaccine Physician for the Northern District of the Northern Liberties, on the 20th of Eighth month (August) last, I have vaccinated nine hundred and fifty persons.

Of these, five hundred and seventy-five had not been previously vaccinated, and three hundred and seventy-five were revaccinations, chiefly adults.

Of the five hundred and seventy-five first vaccinations, five hundred and thirty-seven were successful, and thirty-eight cases failed—being a failure of about 7 per cent. Of the successful cases, two hundred and thirty-eight were males, and two hundred and ninety-nine were females.

Of the three hundred and seventy-five revaccinations, two hundred and thirty-five were successful, and one hundred and forty failed—being a failure of about 37 per cent. Of the successful cases, sixty-nine were males, and one hundred and fifty-six were females.

The whole number of successful cases is seven hundred and seventy-two.

Of these, 161 were under one year of age.
" 110 " between one and two years.
" 165 " between two and five years.
" 98 " between five and ten years.
" 125 " between ten and twenty years.
" 113 " over twenty years of age.

Previous to the existence of the epidemic small-pox in our midst, the failures among the first vaccinations were about ten per cent., during the past month not more than two per cent., and of fifty cases revaccinated previous to the existence of the epidemic, only three took the disease satisfactorily; and I then felt disposed to condemn the practice as useless; but at present the number of successful revaccinations, as shown above, is so great, as to leave no doubt of the propriety of the measure, and to confirm the view that revaccination ought to be resorted to by all, when the small-pox rages as an epidemic, as it now does, in our city. As the susceptibility to the infection is greatly increased by such an epidemic influence, under existing circumstances, the only probable means of arresting the prevalence of this loathsome disease is, for the whole community to be revaccinated, which is the only safe test of the protection of the system.

All of which is respectfully submitted.

Henry T. Child, M. D.
Vaccine Physician, N. D. N. L.

*Northern Liberties,* First month 1st, 1846.

